# Early Surgical Repair of Bile Duct Injuries following Laparoscopic Cholecystectomy: The Sooner the Better

**DOI:** 10.1055/s-0039-1697633

**Published:** 2019-10-18

**Authors:** Muharrem Battal, Pinar Yazici, Ozgur Bostanci, Oguzhan Karatepe

**Affiliations:** 1 Department of General Surgery, Sisli Hamidiye Etfal Training and Research Hospital, General Surgery Clinic, Sisli, Istanbul, Turkey; 2 Department of General Surgery, Memorial Hospital, General Surgery Clinic, Sisli, Istanbul, Turkey

**Keywords:** cholelithiasis, laparoscopic cholecystectomy, hepato-pancreato-biliary surgery, postoperative complication, bile duct injury, emergency surgery, surgical repair

## Abstract

**Background** We aimed to investigate the outcomes of the immediate surgical repair of bile duct injuries (BDIs) following laparoscopic cholecystectomy.

**Materials and Methods** Between January 2012 and May 2017, patients, who underwent immediate surgical repair (within 72 hours) for postcholecystectomy BDI, by the same surgical team expert in hepatobiliary surgery, were enrolled into the study. Data collection included demographics, type of BDI according to the Strasberg classification, time to diagnosis, surgical procedures, and outcome.

**Results** There were 13 patients with a mean age of 43 ± 12 years. Classification of BDIs were as follows: type E in six patients (46%), type D in three patients (23%), type C in two (15%), and types B and A in one patient each (7.6%). Mean time to diagnosis was 22 ± 15 hours. Surgical procedures included Roux-en-Y hepaticojejunostomy for all six patients with type-E injury, primary repair of common bile duct for three patients with type-D injury, and primary suturing of the fistula orifice was performed in two cases with type-C injury. Other two patients with type-B and -A injury underwent removal of clips which were placed on common bile duct during index operation and replacing of clips on cystic duct where stump bile leakage was observed probably due to dislodging of clips, respectively. Mean hospital stay was 6.6 ± 3 days. Morbidity with a rate of 30% ( *n* = 4) was observed during a median follow-up period of 35 months (range: 6–56 months). Mortality was nil.

**Conclusion** Immediate surgical repair of postcholecystectomy BDIs in selected patients leads to promising outcome.

Laparoscopic cholecystectomy (LC) is considered as the gold standard treatment for patients with symptomatic cholelithiasis. This procedure is among the most commonly performed operations at hospitals where abdominal surgery is applied. Bile duct injury (BDI), which may occur during cholecystectomy procedure, has increased since the introduction of LC. It may lead to life-altering complications resulting in significantly increased mortality and morbidity. 1 The rate of bile duct injury secondary to laparoscopic cholecystectomy is 0.2 to 0.9%. 2 3 A major mode of ductal injury is diathermy burns, which may initially go unnoticed, and usually involve the right or common hepatic ducts.

The experience of the center and the surgeon is most prominent factor for the outcome. Besides, timely diagnosis and appropriate treatment also play great importance in the management of this complex, devastating complication. However, timing of surgical treatment is still a matter of discussion and no satisfactory study has yet been reported to enlighten this issue. 3 This study aimed to investigate the outcome of the patients who underwent immediate (<72 hour) surgical repair due to postcholecystectomy bile duct injury.

## Patients and Methods

Between January 2012 and May 2017, patients who underwent early surgical repair (<72 hour) of postcholecystectomy BDI at Sisli Hamidiye Etfal Training and Research Center Training and Research Hospital, General Surgery Clinic, were reviewed. Institutional review board approved this research (609/2016). Patients referred from other health centers to our hospital were also included. Those with intraoperatively diagnosed and repaired BDI ( *n* = 3), and patients who were managed with interventional techniques ( *n* = 4) or underwent surgical repair in the late period ( *n* = 15) were not included into the study. Retrospective data collection included demographic features of patients, time to diagnosis, classification of injury, type of surgical repair, and postoperative period.

**Fig. 1:**
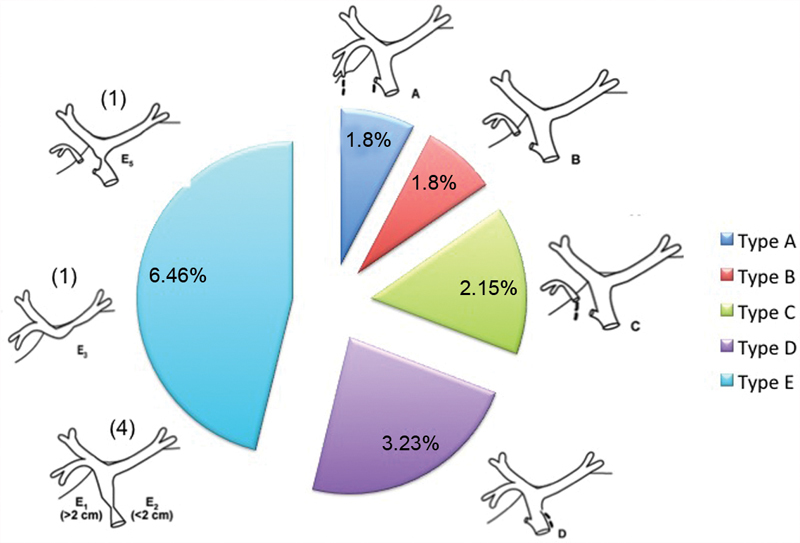
A graphical overview of bile duct injuries. Data labels present the number and percentages of the injury types, respectively.

Preoperative assessment included blood test analyses (a complete blood count [CBC], electrolytes, liver function tests [LFTs], and coagulation studies) and diagnostic methods (abdominal ultrasonography [aUS] and magnetic resonance cholangiopancreatography [MRCP]). Classification of BDIs was made according to the Strasberg classification 4 ( Fig. 1 ).

An experienced team in hepato-pancreato-biliary (HPB) surgery performed all surgical procedures. Criteria for immediate surgical repair of BDI are determined as follows: (1) radiological diagnosis of bile duct injury, (2) diagnosis within initial 72 hours following laparoscopic cholecystectomy, (3) high-output biliary fistula (>500 mL/day), (4) radiological determination of total obstruction of biliary tract, and (5) findings suggestive of biliary peritonitis. Exclusion criteria were as follows: (1) LC due to acute cholecystitis, (2) BDI diagnosed and treated at the time of LC, (3) patients who met the criteria for systemic inflammatory response syndrome, (4) detection of concomitant arterial injury.

## Results

Total incidence of BDI following cholecystectomy procedures in our clinic was 0.08% (18/2,236). Immediate surgical repair (5–72 hour) was performed in 13 (37%) of 35 patients who were diagnosed with iatrogenic BDI. Among 13 patients with early repaired BDI (nine females and four males), seven patients were performed LC in our hospital, while remaining six patients were referred from other hospitals. Mean age was 43 ± 12 years.

**Fig. 2:**
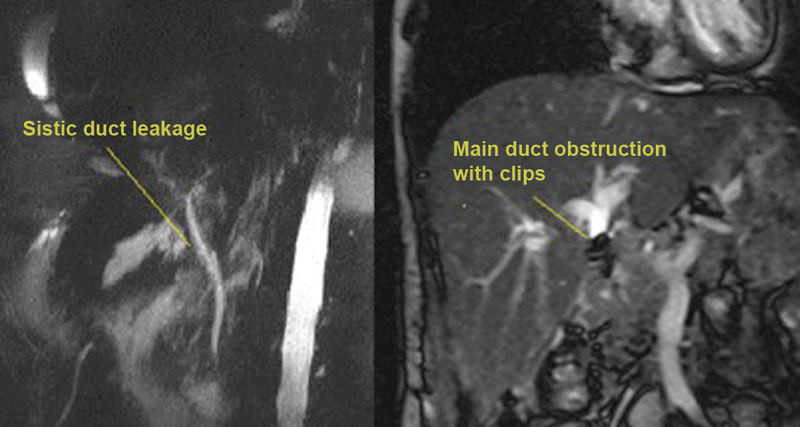
Images of type-A and type-B bile duct injuries.

Diagnostic imaging methods including aUS and MRCP were performed in all patients ( Fig. 2 ). Mean time to diagnosis was 22 ± 15 hours. According to Strasberg's classification, most commonly diagnosed injury was type-E injury (46%, *n* = 6). Clinical findings, surgical procedures, and postoperative detailed data are shown in Table 1 . Biliary peritonitis and fistula in those with a drainage catheter placed during surgery were the most common clinical findings that led to diagnosis. Mechanical icterus was also observed in patients whose bile duct was clipped during the index operation.

**Table 1:** Demographic features, surgical and postoperative data of the patients

	Age (y)	Gender	Findings	Surgical procedure	Type of injury b	Time to diagnosis (h)	Length of hospital stay (d)	Complications
1	38	F	Biliary peritonitis	Roux-en-Y HJ	E2	6	7	–
2	46	F	Biliary fistula	Primary repair	D	8	3	–
3	52	M	Biliary fistula	Roux-en-Y HJ	E3	24	12	Biliary fistula
4	24	F	Mechanical icterus	Removal of clips	B	48	5	–
5	33	F	Biliary peritonitis	Suturing the fistula orifice on gallbladder bed	C	24	3	–
6	65	F	Biliary peritonitis	Roux-en-Y HJ	E1	12	10	SSI
7	47	F	Biliary fistula	Laparoscopic re-clipping the cystic duct	A	36	7	–
8	52	M	Biliary fistula	Primary repair	D	12	5	–
9	37	M	Biliary fistula	Primary repair	D	5	3	–
10	55	F	Biliary peritonitis	Roux-en-Y HJ	E2	48	11	Pneumonia
11	41	F	None a	Roux-en-Y HJ	E1	12	5	–
12	53	M	None a	Roux-en-Y HJ	E5	36	7	–
13	19	F	Biliary peritonitis	Primary repair	C	24	9	Bile leak

Abbreviations: F, female; HJ, hepaticojejunostomy; M, male; SSI, surgical site infection.

a Intraoperatively diagnosed and referred to our hospital without any intervention.

b Strasberg’s classification system was used.

During operative observation, concomitant vascular injury was not detected. Conventional Roux-en-Y hepaticojejunostomy without an intra-anastomotic stent was performed in all patients with type-E injury. Primary repair of common BDI was performed for type-D injury. One patient diagnosed with leakage from gallbladder bed (type-C injury) and another biliary injury to the aberrant right hepatic duct underwent primary suture repair of the biliary fistula. During intraoperative observation in the patient with type-B injury, clips were found on common bile duct. Only removal of clips was performed. Surgical exploration was then completed since recheck of the operating site revealed no other problem and cholangiogram showed patent biliary tree. The last case with type-A injury was revised laparoscopically. Only reclipping of the cystic duct due to dislodged clips was performed. Extensive surgery, such as hepatectomy, was not performed in any patients. Mean hospital stay was 6.6 ± 3 days.

Postoperative complications were observed in four cases (30%). In one patient with type-E injury, who underwent hepaticojejunostomy, low-output biliary fistula was developed. Likewise, another postoperative biliary fistula was observed in a patient with type-C injury which had been treated using primary repair. Both were resolved spontaneously within 15 and 26 days, respectively. Surgical site infection ( *n* = 1) and pneumonia ( *n* = 1) were among other complications. In a median follow-up period of 35 months (range: 6–56 months), no biliary stricture or mortality was observed.

## Discussion

Definitive studies comparing methods to minimize or manage biliary tract complications following laparoscopic cholecystectomy are unlikely to be performed, because BDIs are relatively infrequent. Small series limit the opportunity to draw strong conclusions. The present study is limited by a small sample size; however, it highlights the fact that immediate repair of BDI may ensure promising outcome with improved patient safety and reduced cost.

Laparoscopic cholecystectomy is one of the most commonly performed surgical procedures all over the world. It is defined as the “gold standard” treatment for patients with symptomatic cholelithiasis or acute cholecystitis, if not contraindicated. Development of BDI following LC is a rare but severe complication. 5 Although LC is the most preferred surgical method for treatment, it has higher rates for risk of BDI (2–4-fold increased risk) when compared with open cholecystectomy. 6 Variable anatomy of bile ducts, inexperience of surgeons in HPB surgery and previous or ongoing inflammation at the time of cholecystectomy are among risk factors for development of BDI following cholecystectomy. 7 8 Additionally, contracted gallbladder, unexpected bleeding requiring application of excessive number of clips, or challenging dissection of Calot's triangle, Rouviere's sulcus, median umbilical fissure, and hepatic artery are among other reasons that may give rise to BDI intraoperatively. 9

Surgical management of BDI sustained during LC improves the outcome. However, delay in recognition and treatment leads increased morbidity rates due to severe episodes of cholangitis, jaundice, and intraabdominal sepsis. 10 Even in the case of an early diagnosis, most centers wait 6 to 8 weeks for inflammation to subside and allow the poor status of patients to recover. A delay also allows the biliary ducts to become more dilated which ensures conduction of uncomplicated anastomosis. Thus, early identification and repair are a life-saving approach. 11 The rate of intraoperative diagnosis of BDI is approximately 35%. 12 In our series considering both referred patients and those with an index surgery in our clinic; the rate of intraoperative diagnosis was 20% (6/31). Most of the patients are diagnosed in the postoperative period. Findings in patients with suspected postoperative BDI includes deterioration of patients' clinical picture, elevation of serum bilirubin levels, and intra-abdominal fluid collection revealed by ultrasonography. In case of suspicion for any injury, intraoperative diagnostic procedures, such as cholangiography should be performed. It is better to be evaluated by a surgeon experienced in HPB surgery and a radiologist, if needed. 8 Carroll et al reported a higher rate of surgical success in surgical repair of BDIs by a reference center for HPB surgery when compared with those performed by primary surgeon with a success rate of 27%. 13 In our clinic approach, we routinely perform MRCP, in case of suspicion, and Endoscopic retrograde cholangiopancreatography (ERCP), if necessary.

Time to diagnosis, severity of BDI, patient's health status and accessibility to HPB surgical centers have an important role for determining treatment algorithm. The purpose of surgical repair is to ensure a patent biliary system and avoid complications, such as biliary fistula, intra-abdominal abscess, biliary stricture, recurrent cholangitis and secondary biliary cirrhosis. 14 In this study, all patients were managed in the first 72 hours postcholecystectomy. Considering biliary system-related complications, low-output biliary fistula, which resolved spontaneously, was observed only in one patient (7.6%). Although certain bile leaks can be managed by applying stent and endoscopic sphincterotomy, surgical approach may be needed in case of severe injuries. 15 16 Endoscopic sphincterotomy, nasobiliary stent insertion, and biliary stenting, or combinations of these methods are among treatment options. 17 18 Weber et al stated that endoscopic treatment was successful in those with peripheral bile duct leaks, while lower success rate was reported for common BDIs. 19 Chow et al applied endoscopic sphincterotomy and nasobiliary drainage on patients with bile leakage; authors stated that this procedure can only be successful on acute and noncomplicated cases. 20 As seen in both studies, endoscopic treatments can reach higher success rates, especially injuries are mostly recognized in treatment of minor BDIs.

Timing of reconstructive surgery is one of the most important factors that can affect outcome of treatment approach. 21 It is a matter of debate. Initial 48 to 72 hours is the time of the ending of inflammation phase and beginning of proliferation phase in wound healing. In the meantime, fibrosis also begins. And after this period, surgical repair of BDI has a high-stricture rate. 22 It is considered that if diagnosis is made during surgical procedure, reconstructive surgery applied simultaneously can conclude rather successfully, with low morbidity and mortality rates. 23 24 Several studies show that patients who undergo operation in the acute phase present with higher rates of perioperative and postoperative complications than patients operated in a delayed phase. 23 25 However, Felekouras et al, who stressed the importance of experience in HPB surgery, suggested that early reconstruction of BDI (<2 weeks) was as safe as late reconstruction. 26 In a comparative study between early and late repair, Fischer et al reported less intra-abdominal abscess and shorter length of hospital stay in patients who underwent surgical repair during initial 72 hours compared with late repair. 27 Sahajpal et al recommended reoperation within initial 72 hours, otherwise 6 weeks later. 21

One of the most important factors in the management of patients with BDI is the experience of surgical team. 27 Several recent studies showed that early repair by an HBS was the superior strategy for the treatment of BDI in properly selected patients, 21 28 29 while some authors have not recommended. 30 When BDI is suspended intraoperatively, injury repair is usually performed during index operation in surgical centers where a surgical team experienced in HPB surgery is available. It has been already established that outcome of surgical repair performed within initial 72 hours is comparable to the results when injury is repaired at the time of diagnosis during index surgery. Felekouras et al also showed equal long-term outcomes when an HPB specialist performed the surgical repair in early period compared with late reconstruction. 26

Limitations of our study included the lack of a control group, a small sample size, and retrospective design of the study.

## Conclusion

In conclusion, early surgical procedures performed in every selected cases may result in lower morbidity and mortality rates. However, such procedures are required to be performed at specialized HPB surgical centers.
